# Epigenetic effect of testosterone in the behavior of *C. elegans*. A clue to explain androgen-dependent autistic traits?

**DOI:** 10.3389/fncel.2014.00069

**Published:** 2014-03-04

**Authors:** M. Mar Gámez-Del-Estal, Israel Contreras, Rocío Prieto-Pérez, Manuel Ruiz-Rubio

**Affiliations:** Departamento de Genética, Universidad de Córdoba, Hospital Universitario Reina Sofía, Instituto Maimónides de Investigación Biomédica de CórdobaCórdoba, Spain

**Keywords:** testosterone, *Caenorhabditis elegans*, epigenetics, gentle touch, pharyngeal pumping, nhr-69, nuclear hormone receptor, Autism spectrum disorders (ASDs)

## Abstract

Current research indicates that the causes of autism spectrum disorders (ASDs) are multifactorial and include both genetic and environmental factors. To date, several works have associated ASDs with mutations in genes that encode proteins involved in neuronal synapses; however other factors and the way they can interact with the development of the nervous system remain largely unknown. Some studies have established a direct relationship between risk for ASDs and the exposure of the fetus to high testosterone levels during the prenatal stage. In this work, in order to explain possible mechanisms by which this androgenic hormone may interact with the nervous system, *C. elegans* was used as an experimental model. We observed that testosterone was able to alter the behavioral pattern of the worm, including the gentle touch response and the pharyngeal pumping rate. This impairment of the behavior was abolished using specific RNAi against genes orthologous to the human androgen receptor gene. The effect of testosterone was eliminated in the *nhr-69* (*ok1926*) deficient mutant, a putative ortholog of human *AR* gene, suggesting that this gene encodes a receptor able to interact with the hormone. On the other hand the testosterone effect remained in the gentle touch response during four generations in the absence of the hormone, indicating that some epigenetic mechanisms could be involved. Sodium butyrate, a histone deacetylase inhibitor, was able to abolish the effect of testosterone. In addition, the lasting effect of testosterone was eliminated after the dauer stage. These results suggest that testosterone may impair the nervous system function generating transgenerational epigenetic marks in the genome. This work may provide new paradigms for understanding biological mechanisms involved in ASDs traits.

## Introduction

Autism spectrum disorders (ASDs) are diagnosed with a ratio of about four (male): one (female) across the whole spectrum (Baird et al., 2006), increasing to eight or nine to one in samples with higher functioning patients (Mandy et al., 2011). Some studies suggest that factors in neonatal development, such as those associated with male vs. female sexual development, may play a role in the etiology of some forms of this psychopathology (Baron-Cohen et al., 2003; Keller and Ruta, 2010). Thus, the prenatal environmental has been proposed as a key factor in the connection between autism and maleness. In 1973 it was observed that testosterone neonatal exposure controls male synaptic features in the rat hypothalamus (Raisman and Field, 1973). Subsequent studies have confirmed the effect of perinatal androgen hormone in the masculinization of brain morphology and function in rats, mice, and other mammalian animal models (Morrison and Rieder, 2004). In particular, the role of fetal testosterone has been highlighted as an influential hormone on cognitive and psychological brain development (Baron-Cohen et al., 2004). There are studies supporting this assumption indicating that testosterone exposure has permanent effects on brain development that may guide to male-differences, cognition and behavior respect to females (Cohen-Bendahan et al., 2005a,b; Hines, 2008; Auyeung et al., 2009; Whitehouse et al., 2012; Saenz and Alexander, 2013; Teatero and Netley, 2013). Therefore, it has been hypothesized that high levels of testosterone during early development might be a risk factor for ASDs. This idea is supported by several studies showing that testosterone levels are associated with autistic-like traits (Auyeung et al., 2009, 2010, 2012). Further, the importance of sex steroid related genes in ASDs is sustained by studies reporting associations between polymorphisms in genes involved in sex steroid synthesis/metabolism and ASDs and autistic-like traits (Chakrabarti et al., 2009; Henningsson et al., 2009; Zettergren et al., 2013). Children of mothers affected by hyperandrogenic polycystic ovary syndrome seem to have a higher risk for ASDs probably due to an unbalanced prenatal exposure to high levels of testosterone (Palomba et al., 2012). Finally, other results have shown association between the androgen receptor (*AR*) gene and ASDs (Henningsson et al., 2009).

The human *AR* gene, also known as *NR3C4* (nuclear receptor subfamily 3, group C, member *4*), is a nuclear receptor activated by binding either one of the two androgenic hormones, testosterone, or dihydrotestosterone (Roy et al., 1999). The hormones bind in the cytoplasm to the receptor which then translocate into the nucleus to act upon transcription (Brinkmann, 2011). During androgen-dependent gene activation, histone demethylases are involved in the control of gene expression (Metzger et al., 2006). Specifically, phosphorylation of histone H3 at threonine 6 by protein kinase C beta-1 (PRKCB1) appears to prevent lysine-specific demethylase 1 from demethylating histone H3 at lysine 4 (H3K4) during androgen receptor-dependent gene activation (Metzger et al., 2010). The down-regulation of *PRKCB1* in the temporal lobe has been correlated with ASDs (Lintas et al., 2009). Therefore, there is a strong current of opinion that considers the involvement of epigenetic mechanisms in autism (Mbadiwe and Millis, 2013).

Although studies in patients with ASDs have contributed significantly to the understanding of the pathogenesis of these diseases, many aspects of the molecular etiological basis remain unknown. Mammalian animal models have obvious advantages in order to translate them to humans, but their neuronal wiring maps are highly complex. The nematode *Caenorhabditis elegans* is an organism that has some exceptional characteristics for studying behavior and neurological diseases (Brenner, 1974; Calahorro and Ruiz-Rubio, 2011; Bessa et al., 2013). Up to the present time, only the neuronal wiring diagram of this nematode has been determined (White et al., 1986; Jarrell et al., 2012).

Steroid hormone receptors are included in the large superfamily of nuclear hormone receptors (NHRs), a group of transcription factors that bind lipophilic hormones (e.g., steroids, retinoids, thyroid hormones, bile-acid like hormones, and fatty acids) and they control the transcription of many target genes. *C. elegans* genome contains at least 284 predicted nuclear receptor gene (Gissendanner et al., 2004). Except the DAF-12 (dauer formation 12), which is the best understood steroid hormone receptor in *C. elegans* at the functional level (Galikova et al., 2010), the other NHRs are “orphan receptors” whose ligands have not been identified yet. On the other hand, the nematode requires dietary cholesterol during all developmental stages (Shim et al., 2002) and it has been reported that the worm has an ecdysteroid-like substance (Mercer et al., 1988). A more recent work showed that *C. elegans* contained several hormonal steroids, including pregnenolone (3β -hydroxy-pregn-5-en-20-one) and other pregnane and androstane derivatives. It has been found that pregnenolone increased the worm lifespan and influenced the regulation of aging. This study suggested that steroid hormones in *C. elegans* are synthesized from cholesterol since they are not detected in adults growing in cholesterol-deprived conditions (Broue et al., 2007). Other studies have described the effect of several vertebrate steroid sex hormones and synthetic hormones on *C. elegans* reproduction (Tominaga et al., 2003; Mimoto et al., 2007). For instance, testosterone at 5 μM reduced fecundity and this effect was significantly higher after long-term exposure to this hormone (Tominaga et al., 2003).

The implication of specific genes in autism, in particular those encoding neuroligins and neurexins, has supported the use of this nematode in the study of ASDs (Calahorro et al., 2009; Hunter et al., 2010). Moreover, behaviors impaired in neuroligin (*nlg-1*) and neurexin (*nrx-1*) mutants of *C. elegans* were rescued by transgenic expression of human orthologous genes *NLGN1* (Calahorro and Ruiz-Rubio, 2012), and alpha- and beta- *NRXN1* isoforms (Calahorro and Ruiz-Rubio, 2013), respectively. These observations revealed that human neuroligin and neurexin were functional in the nematode. In addition, methylphenidate (a dopamine reuptake inhibitor) and fluoxetine (a serotonin reuptake inhibitor), two drugs widely used for the treatment of behavioral disorders in humans, were able to restore behavioral impairments related to dopamine and serotonin pathways in neuroligin deficient mutants of *C. elegans* (Izquierdo et al., 2013).

One of the advantages of studying epigenetic mechanisms caused by testosterone in *C. elegans* could be the absence of DNA methylation in this organism (Bird, 2002), given that it links the possible epigenetic mechanisms exclusively to histone modifications. In this study, we observed that testosterone alters the mechanosensory response to gentle touch and the pharyngeal pumping rate of the worm. This effect of testosterone was abolished in the *nhr-69* (*ok1926*) deficient mutant, a putative ortholog of human *AR* gene, suggesting that this gene encodes a nuclear receptor able to interact with the hormone. The testosterone effect remained in the mechanosensory response during four generations in the absence of the hormone, indicating that some epigenetic mechanisms could be involved. These results suggest that testosterone may impair the nervous system functionality through a specific receptor generating transgenerational epigenetic marks in the genome.

## Materials and methods

### Strains and maintenance

All nematodes were grown and maintained at 20°C under standard conditions on Nematode Growth Medium (NGM) agar plates (Brenner, 1974). Table 1 shows the *C. elegans* strains used in this study. OP50 *Escherichia coli* strain was obtained from the Caenorhabditis Genetic Center (University of Minnesota, Minneapolis, MN, USA). For the bacterial feeding RNA interference assay, HT115 *E*. *coli* strain (DE3) with plasmid pL4440 carrying ORFs from different *C. elegans* genes were used. They were obtained from Julián Cerón, at the Bellvitge Institute for Biomedical Research (IDIBELL, Barcelona, Spain) and from Peter Askjaer at the Centro Andaluz de Biología del Desarrollo (CABD, Sevilla, Spain).

**Table 1:** ***C. elegans* strains used in this study**.

**Strain name**	**Genotype**	**Source**
N2	Wild type, DR subclone of CB original	CGC^a^
TU3335	*lin-15B(n744) X; uIs57 [unc-119p::YFP + unc-119p::sid-1 + mec-6p::mec-6]*	CGC
VC1127	*nhr-126 (gk520) V*	CGC
RB812	*fax-1 (ok624) X*	CGC
RB1578	*nhr-69 (ok1926) I*	CGC
VC1120	*nhr-17 (gk509) X*	CGC
VC469	+/*szT1[lon-2(e678)] I; nhr-25(ok645)/szT1 X*	CGC
STE68	*nhr-49(nr2041) I*	CGC
RB1592	*nhr-64(ok1957) I*	CGC
VC40185	*nhr-226 (gk502851) V*	CGC
CRR300^b^	*nhr-69 (ok1926) I*	This study

a CGC: Caenorhabditis Genomic Center.

b After outcrossing RB1578 strain with N2 wild type two times.

### Behavioral assays

All the behavioral assays experiments were carried out at 20°–24°C with L4 animals.

#### Gentle touch response assay

This assay was performed using an eyebrow hair attached to a toothpick. The phenotype was tested by stroking the worm ten times with the eyebrow hair alternating the anterior (just behind the pharynx) and posterior (just before the anus) part of the body. A positive response causes the animal to move backward or forward respectively (Chalfie et al., 1985; Bounoutas and Chalfie, 2007).

#### Pharyngeal pumping assay

Pumping rates of individual worms were quantified by counting pharyngeal contractions. Individual L4 hermaphrodite grown on NGM plates seeded with OP50 were recorded, focusing on pharyngeal pumping. The video recording was followed by off-line analysis in slow motion.

### Testosterone assays

Testosterone powder (Sigma-Aldrich, St. Louis, MO, USA) was diluted in 70% ethanol to obtain a stock concentration of 100 mM and was added to NGM plates to get a final concentration of 0.01, 0.1, or 1 mM. In the majority of the experiments 1 mM testosterone was used because the worms grew well and we could be sure that the hormone was not a limiting factor. Gravid adults were placed on seeded NGM control plates (with 0.7% ethanol) or seeded NGM with testosterone (1 mM testosterone; 0.7% ethanol) and allowed to lay eggs to ensure that developing embryos were exposed to testosterone. The progeny were allowed to develop to the late L4 larval stage and then assayed for gentle touch response and pharyngeal pumping rate.

### Bacterial feeding RNA interference assays

Wild type N2 Bristol and TU3335 *C. elegans* strains were used for *RNAi* experiments (Table 1). HT115 *E*. *coli* strain (DE3) with plasmid pL4440 carrying *nhr-14* (T01B10.4), *fax-1* (F56E3.4), *nhr-111* (F44G3.9), *nhr-236* (Y38F2AL.5), or *mec-4* (T01C8.7) gene ORFs were provided by Julián Cerón at the Bellvitge Institute for Biomedical Research (IDIBELL, Barcelona, Spain). Worms were fed on standard agar plates supplemented with carbenicillin (50 mg/mL-1) and 1 mM IPTG to induce *dsRNA* production. HT115 transformed with the empty pL4440, pL4440/unc-22 constructs (from Peter Askjaer at the Centro Andaluz de Biología del Desarrollo, CABD, Sevilla, Spain), were also used as controls in the experiments.

### Sodium butyrate assays

Sodium butyrate (SB) powder (Sigma-Aldrich, St. Louis, MO, USA) was dissolved in water to obtain a stock concentration of 100 mM and added to NGM plates to get a final concentration of 1 mM. Gravid adults coming from NGM testosterone plates (1 mM testosterone; 0.7% ethanol) were placed or seeded NGM plates with testosterone and SB (1 mM testosterone; 0.7% ethanol; 1 mM sodium butyrate), and allowed to lay eggs. The progeny were allowed to develop to the late L4 larval stage, and then they were assayed for gentle touch response and pharyngeal pumping rate.

### Dauer stage assays

Gravid adults were placed on NGM control plates (0.7% ethanol) or NGM testosterone plates (1 mM testosterone; 0.7% ethanol) and allowed to lay eggs. The progeny were allowed to develop to the dauer larval stage (one month approximately). Then, dauer larval stage animals were placed on NGM plates seeded with OP50 bacteria and allowed to develop to the late L4 larval stage. Then, the animals were assayed for gentle touch response and pharyngeal pumping rate.

### Transgenerational assay

Gravid adults were placed on seeded NGM control plates (0.7% ethanol) or seeded NGM testosterone plates (1 mM testosterone; 0.7% ethanol) and allowed to lay eggs. The progeny were allowed to develop until the late L4 larval stage or to the gravid adult stage. The L4 animals were assayed for gentle touch response and pharyngeal pumping rate. The gravid adult animals were placed on seeded NGM plates without testosterone and allowed again to develop to the late L4 larval stage or to the gravid adult stage. Again, the L4 animals were assayed for gentle touch response and pharyngeal pumping rate, and the gravid adult animals were placed on seeded NGM plates without testosterone and allowed again to develop to the late L4 larval stage or to the gravid adult stage. This procedure was successively repeated to analyze the behavior in the following generations without testosterone.

### Statistical analysis

Comparisons shown in each experiment were done by One-Way ANOVA using Excel statistical tool.

## Results

### Influence of testosterone in gentle touch response and pharyngeal pumping rate

**Figure 1:**
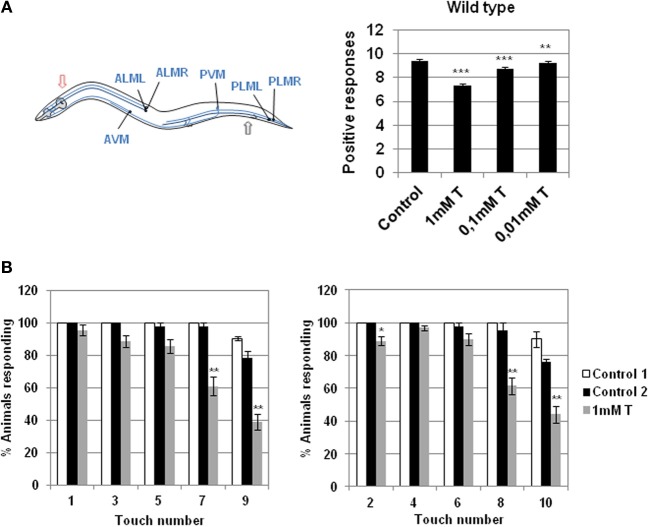
**Effect of testosterone on gentle touch response. (A)** Left panel: diagram of 6 mechanosensory neurons involved in gentle touch response (blue) modified from Bounoutas and Chalfie (2007). In the gentle touch response animals were touched by stroking an eyebrow hair across the body just behind the pharynx (red arrow) for the anterior touch response, or just before the anus (black arrow) for the posterior touch response; Right panel: gentle touch response of N2 wild type strain was carried out either in the presence or absence of different concentration of testosterone (0.01, 0.1, and 1 mM). Data were analyzed as number of positive responses to ten alternative gentle touches in the anterior and the posterior part of the body. **(B)** Testosterone alters the wild type gentle touch response. In the absence of the hormone N2 wild type strain responds five times consecutively to gentle touch in the anterior and posterior part of the body. In the presence of testosterone (1 mM) the worm fails to respond significantly to the fourth and the fifth touch. Data were quantified as percentage of animals responding to ten alternative gentle touches in the anterior (left panel) and the posterior (right panel) part of the body (five each). At least three independent experiments were carried out (at least 10 L4 worms per experiment). Bars represent the mean ± SEM. Statistical significance was calculated by 1-factor-ANOVA. Statistical *p*-values in **(A)**: ^***^p < 0.01, ^**^p < 0.001 vs. “Control.” Control: NGM + 0.7% ETOH; T: NGM + 0.7% ETOH + 1, 0.1, or 0.01 mM testosterone. Statistical *p*-values in **(B)**: ^**^p < 0.01, ^*^p < 0.05 *vs*. “Control 2.” Control 1: NGM; Control 2: NGM + 0.7% ETOH; 1 mM T: NGM + 0.7% ETOH + 1 mM testosterone.

When the nematode receives a tactile stimulus with an eyebrow hair in the anterior or posterior part of its body, it changes the direction of motion inducing movement back or forward respectively (Chalfie et al., 1985). Sensory cells of *C. elegans* translate mechanical inputs into ionic currents, which activate a neural circuit that drives a locomotory response (O'Hagan et al., 2005). The presence of different concentrations of testosterone induced the loss of a significant capability of the mechanosensory response with respect to the wild type strain (Figure 1A).

When *C. elegans* is stimulated repeatedly with an eyebrow hair (gentle touch), the stimulus fails to produce a response and the animal become refractory (Chalfie et al., 1985). Figure 1B shows that in the absence of testosterone the wild type strain responds five times consecutively to gentle touch in the anterior and posterior parts of the body, whereas in the presence of testosterone the worms failed to respond mainly to the fourth and fifth touch.

**Figure 2:**
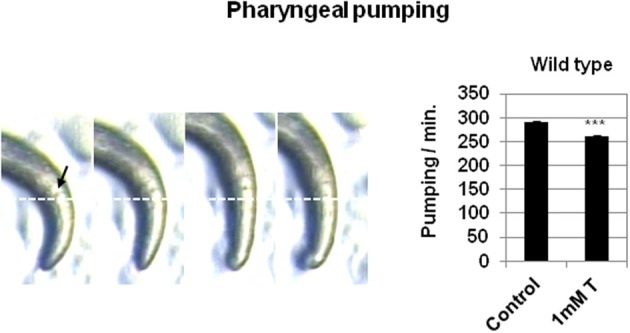
**Testosterone alters the pharyngeal pumping rate. Left panel**: Pharyngeal pumping images captured by a stereomicroscope (8× magnification). White line was used as reference to measure grinder (black arrow) movement. **Right panel**: pharyngeal pumping rate analysis of N2 wild type strain in the presence or absence of 1 mM testosterone. At least three independent experiments were carried out (at least 10 L4 worms per experiment). Bars represent the mean ± SEM. Statistical significance was calculated by 1-factor-ANOVA. Statistical *p*-values: ^***^p < 0.001 vs. “Control.” Control: NGM + 0.7% ETOH; T: NGM + 0.7% ETOH + 1 mM testosterone.

We investigate whether testosterone had an effect in other complex behavior where motor control requires the interaction of the nervous system, muscles, and environment. The pharynx muscle movements of the nematode *C. elegans* is very well characterized (Bean et al., 2004). The worm feeding depends on a neuromuscular pump that connects the mouth to the intestine. The pharyngeal muscle takes bacteria and transports them through the gut. This is achieved with a combination of two actions, pumping and isthmus peristalsis. Pumping is the best understood and consists of a cycle of contraction and relaxation that sucks in liquid with suspended particles from the environment, and then expels the liquid trapping the solid particles. Pharyngeal muscle is capable of pumping without nervous system input, but during normal rapid feeding its timing is controlled by two pharyngeal motor neuron types (Avery and Horvitz, 1989). We examined the pharyngeal pumping in the absence and presence of 1 mM testosterone and a significant reduction in the number of pumps count was observed in the presence of the hormone (Figure 2).

### *C. elegans* orthologous genes of the human androgen receptor

To test the presence of orthologous genes of the human androgen receptor gene (*AR*) in the *C. elegans* genome, we carried out a BLAST search using the human AR receptor ligand-binding domain sequence (residues from 690 to 919) as a query, and as database the protein sequences of *C. elegans* (http://blast.ncbi.nlm.nih.gov/). Table 2 shows the results obtained from the BLAST analysis were the *C. elegans* proteins with homology are ordered respect to their E-values. The protein with the lowest E-value, that is, the best matching with the human AR receptor ligand-binding domain was NHR-69.

**Table 2:** Sequence comparison of the human androgen receptor ligand-binding domain (hAR-LBD) and the proteins in the *C. elegans* database ^*^.

**Protein symbol**	**Accession number**	**Protein size (residue number)**	**LBD position**	**Alignment position**	**% identity**	***E*-value**
NHR-69	NP_492615.2	373	98–340	133–282	24.84	3,00E-06
NHR-91	NP_001024953.1	474	222–465	248–418	23.26	3,00E-04
NHR-192	NP_505584.2	352	160–321	180–348	21.71	6,00E-04
S/T/R NHR homolog nhr-35	T34093	515	230–441	244–274	45.16	0,03
NHR-17	NP_510115.1	396	182–360	192–358	25.74	0,03
NHR-35	NP_001024366.1	486	174–378	188–307	28.57	0,039
NHR-25	NP_001024551.1	492	269–421	274–314	36.59	0,086
NHR-49	AAG15140.1	337	59–253	60–170	24.14	0,15
FAX-1	NP_001263953.1	390	233–367	235–320	23.42	0,21
NHR-47	NP_504455.1	579	385–522	208–267	25	0,55
NHR-46	NP_001023065.1	576	365–540	372–411	33.33	0,83
NHR-248	NP_507334.1	342	149–313	235–342	27.19	0,83
NHR-64	NP_001021043.2	344	101–323	128–269	20.39	1,1
NHR-10	AAO39172.1	396	197–355	200–231	37.5	1,2
NHR-119	NP_494368.2	708	213–382	213–256	27.27	2,3
NHR-219	NP_505314.1	511	289–492	300–330	29.03	3,7
NHR-226	NP_503630.1	387	165–368	174–232	28.81	3,7
NHR-126	NP_503609.2	404	184–388	193–227	28.57	8,1

* http://blast.ncbi.nlm.nih.gov/

A selection of worm proteins with the highest identity values is shown. The results were obtained from BLAST analysis using human hAR-LBD (residues from 690 to 919) as a query.

On the other hand the proteins obtained with the BLAST search were structurally compared with the human AR binding with testosterone (Table 3). The quality of the model was measured using QMEAN (Benkert et al., 2008, 2009). The QMEAN score4 measures the global score of the whole model, reflecting the predicted model reliability range from 0 to 1 with higher values for better models. The highest reliability range was 0.48 for the FAX1 protein.

**Table 3:** **Putative genes from *C. elegans* that could act as testosterone receptors**.

**Protein symbol**	**Accession number**	**Protein size (residue number)**	**Modeled residue range**	**QMEAN score4**
FAX-1	NP_001263953.1	390	223–386	0.48
NHR-119	NP_494368.2	708	208–413	0.463
NHR-192	NP_505584.2	352	154–351	0.451
NHR-69	NP_492615.2	373	130–366	0.449
NHR-64	NP_001021043.2	344	97–344	0.442
NHR-126	NP_503609.2	404	185–381	0.426
NHR-35	NP_001024366.1	486	177–384	0.423
NHR-226	NP_503630.1	387	166–362	0.422
NHR-17	NP_510115.1	396	178–390	0.416
NHR-236	CCD66972.1	283	115–273	0.415
NHR-49	AAG15140.1	337	37–256	0.404
NHR-248	NP_507334.1	342	120–341	0.402
NHR-25	NP_001024551.1	492	265–460	0.4
NHR-91	NP_001024953.1	474	234–453	0.389
NHR-111	CAB05521.2	311	141–307	0.384
NHR-219	NP_505314.1	511	290–484	0.378
NHR-46	NP_001023065.1	576	360–574	0.352
S/T/R NHR homolog nhr-35	T34093	515	233–480	0.278
NHR-47	NP_504455.1	579	201–578	0.269

*A selection of worm proteins with the highest identity values obtained from BLAST analysis using human hAR-LBD (residues from 690 to 919) vs. protein sequences of C. elegans database (Table 2), were structurally modeled by comparison with human androgen receptor in complex with testosterone. Swiss-Model Proteomic Serve was implemented. QMEANscore4 reflects the predicted model reliability ranging from 0 to 1 with higher values for better models*.

### Candidate genes for nuclear hormone receptor involved in the response to testosterone

Feeding RNAi is efficient in almost all *C. elegans* cells except neurons (Timmons et al., 2001). Expression of SID-1, a transmembrane protein from the worm is required for systemic RNA interference (RNAi) increases the response of neurons to double-stranded RNA delivered by feeding (Calixto et al., 2010). For that reason, we carried out RNAi feeding experiments with bacteria expressing specific ORFs of some *C*. *elegans nhr*- genes, in both the wild type N2 and the TU3335 strains, this latter expressing SID-1 in neurons.

**Figure 3:**
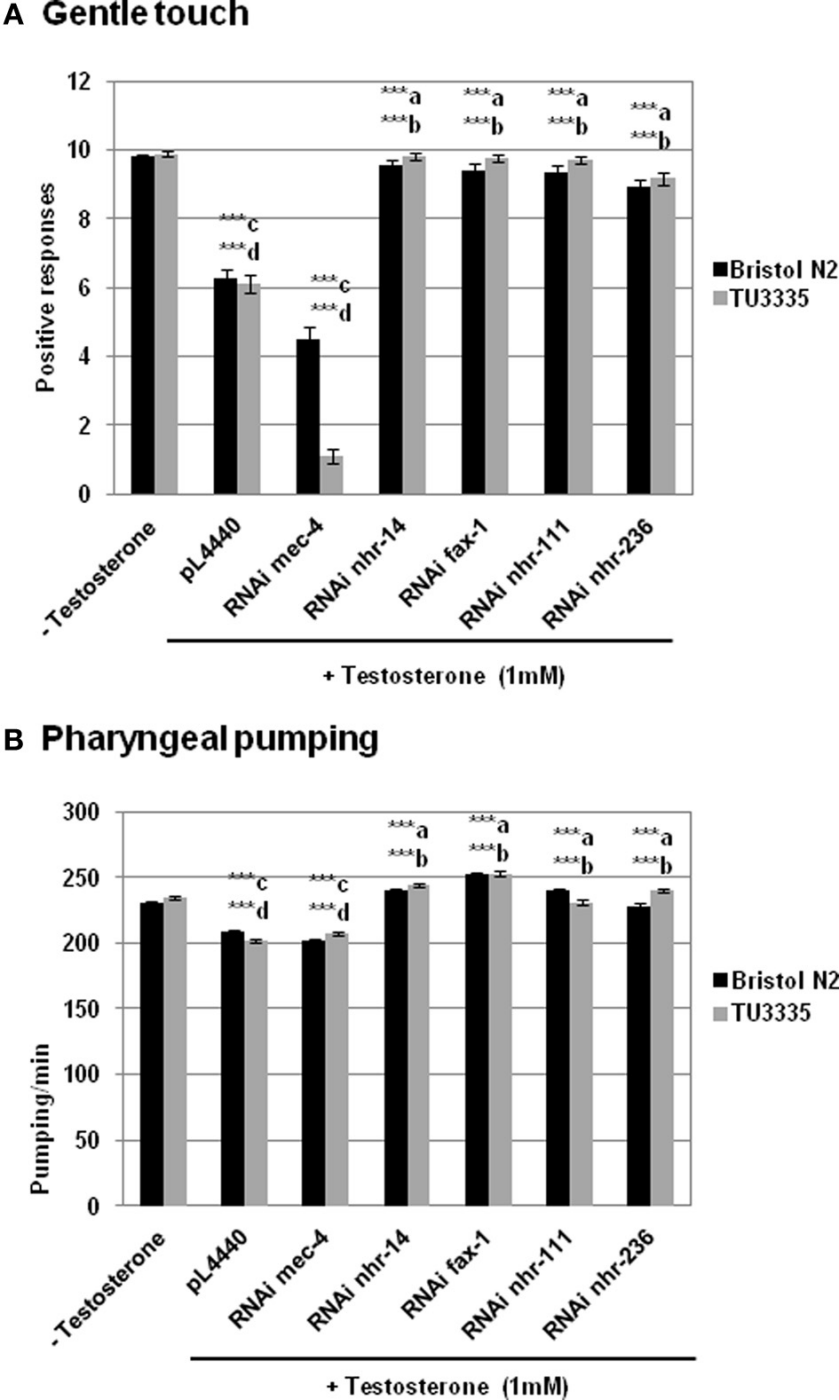
**RNAi feeding against genes from *C. elegans*. orthologous to human androgen and estrogen receptors abolishes the effect of testosterone in the behavior of *C. elegans*.** Bristol N2 and TU3335 (*P*_*unc*−119_*sid* − 1) strains were fed with bacteria carrying the pL4440 vector with different RNAi ORFs of *mec-4*, *nhr-14*, *fax-1*, *nhr-111*, and *nhr-236* genes, or with the empty vector. Gentle touch response **(A)** and pharyngeal pumping rate **(B)** were quantified in the presence or absence of 1 mM testosterone. At least three independent experiments were carried out (at least 10 L4 worms per experiment). Bars represent the mean ± SEM. Statistical significance was calculated by 1-factor-ANOVA. Statistical *p*-values: ^***^a,b *p* < 0.001 *vs*. “pL4440 empty vector” for N2 and TU3335 strains respectively; ^***^c,d *p* < 0.001 *vs*. “−Testosterone” for N2 and TU3335 strains respectively. −Testosterone: NGM + 0.7% ETOH; +Testosterone: NGM + 0.7% ETOH + 1 mM testosterone.

Gentle touch response and pharyngeal pumping rate were analyzed in the absence and presence of testosterone (Figure 3). The results showed that *C. elegans* fed with bacteria containing plasmid pL4440 expressing dsRNA ORFs from *fax-1*, *nhr-111*, and *nhr-236* (all orthologous of the human androgen receptor, see Table 1) showed a recovery in the response to the gentle touch (Figure 3A) and pharyngeal pumping (Figure 3B) assays in the presence of testosterone respect to control fed with bacteria with empty plasmid. This provides evidence for an *in vivo* function of *nhr*- genes in the response of *C. elegans* to testosterone.

Furthermore there was no significant difference between the knockdown strains and either wild type N2 or TU3335 strains without testosterone, an observation that contrasts with the response to gentle touch in worms fed with bacteria expressing dsRNA to *mec-4*, a gene expressed specifically in neurons and required for the gentle touch response (O'Hagan et al., 2005). These results suggest that the deficiency of *nhr*- genes in the effect of testosterone in gentle touch and pharyngeal pumping probably depends on the expression of these genes in different type of cells.

On the other hand the RNAi feeding experiment was also carry out with bacteria containing plasmid pL4440 expressing dsRNA ORFs from *nhr-14*, a *C. elegans* gene ortholog to human estrogenic receptor (Mimoto et al., 2007). The sequence of the protein encoded by this gene presents a region with specific identity to the human androgen ligand-binding domain (12.17%). The results obtained with *nhr-14* knockdown animals were similar to those of *fax-1*, *nhr-111*, and *nhr-236* knockdown ones (Figure 3). One explanation to this observation is an effect of cross-interference due to the high percentage of similarity between all these sequences. In fact, it has been demonstrate that siRNAs may cross-react with targets of limited sequence similarity (Jackson et al., 2003). Cross-interference seems to be very likely if there is 80% nucleotide identity over 200 bp (Kamath and Ahringer, 2003).

### NHR-69 a putative testosterone receptor in *C. elegans*

**Figure 4:**
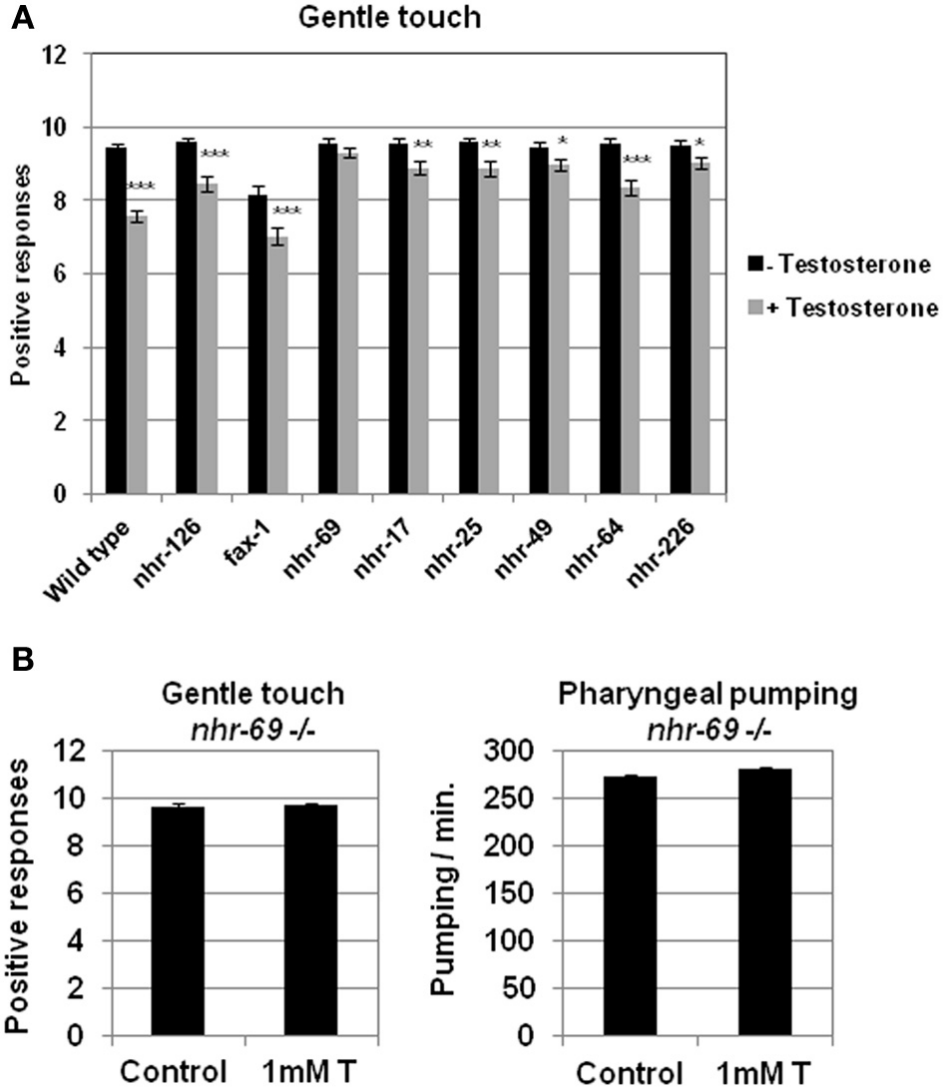
**Screening for testosterone response in different *C. elegans* strains harboring deletions in genes orthologous of human androgen receptor. (A)** Quantitative assays for gentle touch response were carried out in different strain of *C. elegans* defective in genes orthologous to human AR. **(B)** Touch sensitivity (left panel) and pharyngeal pumping rate (right panel) were quantified in *nhr-69*(*ok1926*) defective mutant strain (outcrossed 2×). At least three independent experiments were carried out (at least 10 L4 worms per experiment). Bars represent the mean ± SEM. Statistical significance was calculated by 1-factor-ANOVA. Statistical *p*-values in **(A)**: ^*^p < 0.05, ^**^p < 0.01, ^***^p < 0.001 *vs*. “Wild type + Testosterone”. -Testosterone: NGM + 0.7% ETOH; +Testosterone: NGM + 0.7% ETOH + 1 mM testosterone. Statistical *p*-values in **(B)**: not significant (*p* > 0.05) *vs*. “Control”. Control: NGM + 0.7% ETOH; 1 mM T: NGM + 0.7% ETOH + 1 mM testosterone.

With the objective of finding genes able to function as a testosterone receptor, we carried out a screening for testosterone response in different mutant strains (Table 1) with deletions in genes orthologous of human *AR*. Figure 4A shows the effect of testosterone of these mutants on gentle touch response. Only the strain having a deletion in the gene *nhr-69* did not show response to testosterone. To further confirm this observation, the mutant strain *nhr-69* (*ok1926*) was out-crossed to clean its genome of undesired mutations, and then assayed again with testosterone respect of gentle touch response and pharyngeal pumping rate. The results shown in Figure 4B confirmed that mutant *nhr-69* (*ok1926*) lost the capability to respond to testosterone in both behavioral assays.

### Transgenerational epigenetic inheritance of impaired gentle touch response induced by testosterone

Steroid hormones can induce epigenetic chromatin modifications, including covalent changes of histone proteins, bringing long-lasting adjustments in gene expression in cancer cell lines and peripheral tissues (Ruiz-Cortes et al., 2005; Zhu et al., 2008). There are evidences of epigenetic action of neonatal testosterone in brain masculinization in mice (Murray et al., 2009).

**Figure 5:**
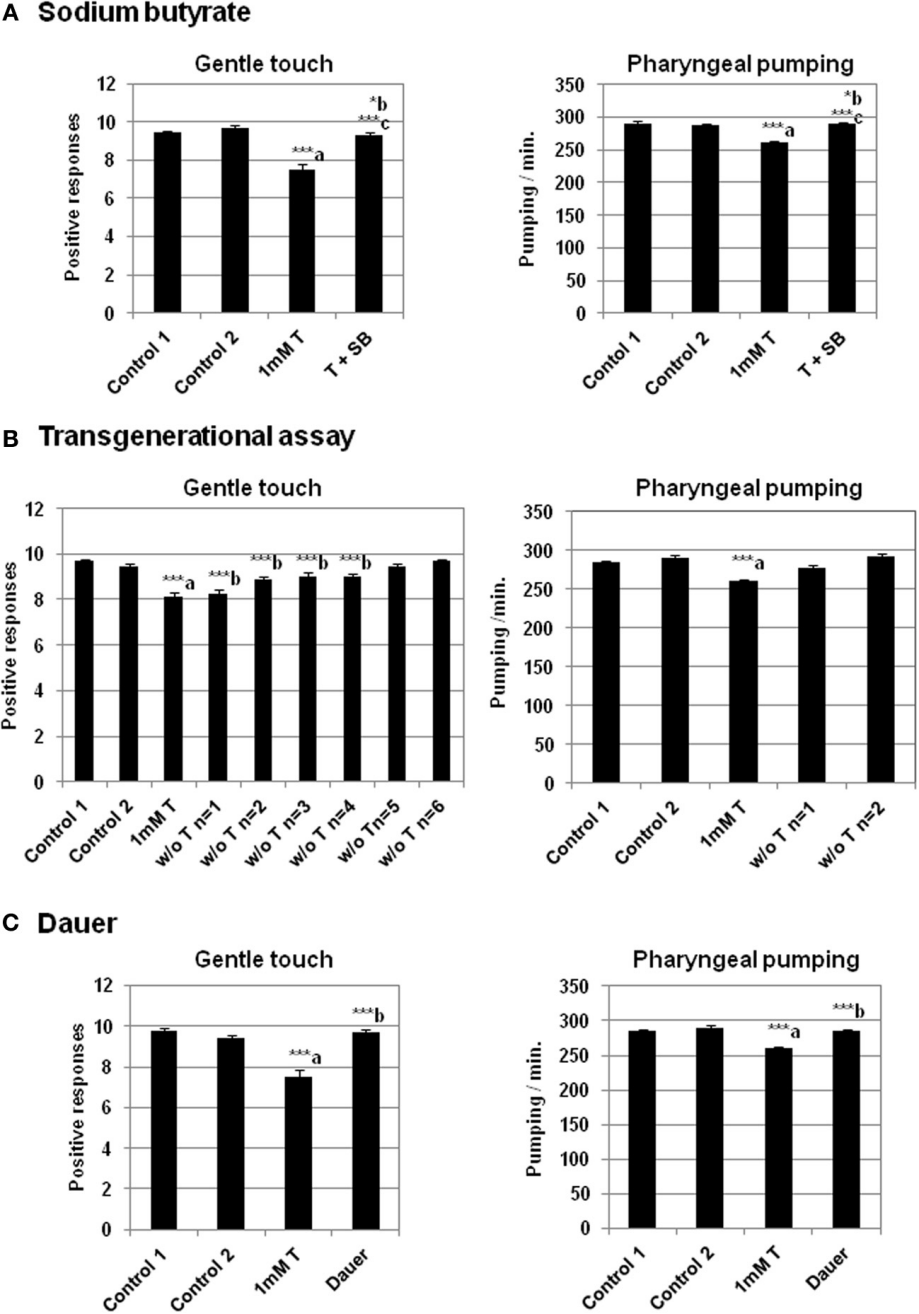
**Sodium butyrate and dauer stage abolish the effect of testosterone in gentle touch response and pharyngeal pumping rate.** Transgenerational epigenetic inheritance of testosterone in *C. elegans*. **(A)** Sodium butyrate (SB) (1 mM) recues impaired gentle touch response (left panel) and pharyngeal pumping rate (right panel) in N2 wild type strain growing in the presence of 1 mM testosterone. **(B)** Transgenerational effect of the testosterone on gentle touch response (left panel) and pharyngeal pumping rate (right panel) in N2 wild type strain after several generations (n) in the absence of testosterone (“w/o T”). **(C)** Analysis of the testosterone effect on gentle touch response (left panel) and pharyngeal pumping rate (right panel) in N2 wild type strain after dauer stage (Dauer). At least three independent experiments were carried out (at least 10 L4 worms per experiment). Bars represent the mean ± SEM. Statistical significance was calculated by 1-factor-ANOVA. Statistical *p*-values in **(A)**: ^***^a *p* < 0.001 *vs*. “Control 1”; ^*^b *p* < 0.05 *vs*. “Control 2”. ^***^c *p* < 0.001 *vs*. “1 mM T”. Control 1: NGM + 0.7% ETOH; Control 2: NGM + 0.7% ETOH + 1 mM sodium butyrate; 1 mM T: NGM + 0.7% ETOH + 1 mM testosterone; T + SB: NGM + 0.7% ETOH + 1 mM testosterone + 1 mM sodium butyrate. Statistical *p*-values in **(B)**: ^***^a *p* < 0.001 *vs*. “Control 2”; ^***^b *p* < 0.001 *vs*. “Control 1”. Control 1: NGM; Control 2: NGM + 0.7% ETOH; 1 mM T: NGM + 0.7% ETOH + 1 mM testosterone. Statistical *p*-values in **(C)**: ^***^a *p* < 0.001 *vs*. “Control 2”. ^***^b *p* < 0.001 *vs*. “1 mM T”. Control 1: NGM; Control 2: NGM + 0.7% ETOH; 1 mM T: NGM + 0.7% ETOH + 1 mM testosterone.

The possible epigenetic changes originated by testosterone in the worm could not be due to DNA methylation, since *C. elegans* has not a predictable DNA methyltransferase in its genome neither 5-methyl cytosine (5 mC) in its DNA (Bird, 2002). This suggests that other mechanisms has to be involved, most likely changes in chromatin histones. Indeed, it has been previously demonstrated that acute administration of sodium butyrate (SB) inhibits histone deacetylase in several organisms, including *C. elegans* (Catoire et al., 2008; Zhang et al., 2009), *Drosophila* (Tie et al., 2009), mammalian cells (Candido et al., 1978; Catoire et al., 2008) and even *Saccharomyces cerevisiae* (Yu et al., 2005) and plants (Chua et al., 2003). Therefore, to determine the possibility of the existence of epigenetic mechanisms induced by testosterone in the worm, we studied whether the histone deacetylase inhibitor SB had any effect on the results observed in the behavior associated to the steroid hormone action. Acute administration of SB resulted in marked increase in acetylation of histone H3 lysine 14 and histone H4 lysine 8 in specific tissues of mice (Itzhak et al., 2013). Figure 5A shows that SB (1 mM) abolishes the effect of testosterone on gentle touch response and pharyngeal pumping rate in *C. elegans*.

To determine whether the testosterone was able to induced epigenetic changes in behavior that were transgenerationally inherited, we studied its action on gentle touch response and pharyngeal pumping over several generations in the absence of the hormone. The results presented in Figure 5B shows that in the case of gentle touch response the effect could still extend in the four subsequent generations. However the reduction of the pharyngeal pumping rate disappeared just in the next generation.

There are mechanisms of reprogramming which are able of erasing epigenetic signatures typified by DNA methylation or histone modification (Apostolou and Hochedlinger, 2013). The life expectancy of *C. elegans* is approximately 2 weeks. However, in conditions of starvation, the developing larvae can adopt an alternative form, called the dauer stage. In this period the worm does not feed and is almost metabolically inactive (Fielenbach and Antebi, 2008). Dauer larva can survive for several months, and prolong the life span of the worm until 12 times more. The observations that the aging clock can be paused in the *C. elegans* dauer stage and reversed by environmental factors, suggest that the causes should be epigenetic mechanisms: on the one hand, blocking the gene expression in the dauer stage by epigenetic marks, and on the other hand reprogramming the metabolism and erasing these marks. For this reason it was possible that the dauer stage, could lead to the removing the epigenetic signals caused by testosterone. As seen in Figure 5C, when the worms were maintained on testosterone until dauer stage, and then growing on plates without the hormone, the gentle touch response and the pharyngeal pumping rate were similar to the control without testosterone.

## Discussion

In humans, testosterone performs critical functions from pregnancy to adolescence. An elevated level of testosterone during early human embryo development has been hypothesized to be a risk factor for ASDs. This idea is supported by several studies showing that high maternal testosterone levels are associated with autistic-like traits in the offspring. The mechanisms by which testosterone interacts with cells and carries out its effects on the development of the nervous system are poorly understood.

This study examines the effect of testosterone in two different behavioral traits of the nematode *C. elegans*, the response to gentle touch stimulation and the pharyngeal pumping rate. The presence of the hormone in the culture media induced the loss of a significant capacity of the reaction to gentle touch stimuli. In fact, it was observed that mechanic stimulation with an eyebrow hair led to a reduction in the response to the fourth and fifth touch of five consecutive touches in both the anterior and posterior parts of the body. Testosterone also induced a decrease in the pharyngeal pumping rate. These behaviors are mediated by distinct neural circuits, suggesting a broad impact of testosterone on neuronal synapse functionality. The way by which testosterone affect the nervous system function is presumably by altering gene expression through a nuclear hormone receptor.

The genome of *C. elegans* contains at least 284 predicted nuclear receptor genes (Gissendanner et al., 2004). This number outnumbers those in mammals (~50 genes) (Zhang et al., 2004). A BLAST search with the human androgen receptor ligand-binding domain sequence against protein sequences of *C. elegans* resulted in several potential orthologous genes. Between them, NHR-69 presented the best matching sequence with the human AR receptor ligand-binding domain. Accordingly with this finding, we observed that the strain RB1578 with a deletion in the gene *nhr-69* (*ok1926*) was insensitive to the effect of testosterone in both behavioral assays.

The gene *nhr-69* was previously defined as being conserved between *C. elegans* and humans (Shaye and Greenwald, 2011). Furthermore, NHR-69 is predicted to function as a transcription factor that could activate or repress transcription in response to a hormonal signal (Gissendanner et al., 2004). A GFP-reporter gene for *nhr-69* was expressed in pharynx, hypodermis, intestine, rectal epithelium, and uterine toroidal epithelial (Gissendanner et al., 2004). In addition a NHR-69::GFP fusion protein driven by the *nhr-69* promoter was detected in the nucleus of the E8 intestinal precursor cells in developing embryos, expression that persisted until adulthood. In adults, expression was also detected in the ASI sensory neurons (gustatory-chemosensory, thermosensory), hypodermis, and tail neurons (Park et al., 2012).

Supporting the results observed in this work, previous *in vitro* experiments demonstrated the capacity of NHR-69 to bind both progesterone and testosterone (Mimoto et al., 2007). More recently it was found that NHR-69 partners with DAF-8. A model is proposed where this interaction represses the *exp-2* gene that encodes a voltage-gated potassium channel. Low EXP-2 increases the secretion of the insulin-like peptide DAF-28 in ASI neurons (Park et al., 2012).

In respect of insulin it has been shown that pan-neuronal expression of APL-1, the *C. elegans* ortholog of the human amyloid precursor protein, disrupts several behavioral traits of the nematode. All these behaviors require activity of the transforming growth factor beta (TGF-beta) signaling pathway and reduced activity of the insulin pathway (Ewald et al., 2012). This might explain, at least indirectly, some of the effect originated by testosterone in behavior, if the binding of NHR-69 to the hormone compete the binding to DAF-8, and therefore modify insulin secretion and the activity of the insulin pathway. It is known that age-dependent neuronal defects are regulated by insulin signaling pathway (Peng et al., 2011).

We also found that the effects of testosterone on behavior were eliminated in presence of the deacetylase inhibitor SB and after dauer stage. Both experimental conditions suggest possible epigenetic mechanisms. In the case of sodium butyrate it is known that inhibits histone deacetylation in mammalian culture cells (Candido et al., 1978) and increases histone acetylation in *C. elegans* (Zhang et al., 2009). More recently was shown that an acute administration of SB resulted in a marked increase in acetylation of histone H3 at lysine 14 and histone H4 at lysine 8 in specific tissues of mice (Itzhak et al., 2013). In respect of dauer larval stage it was demonstrated that transiently passed through this stage, post-dauer adults exhibited significant changes in gene expression profiles and chromatin states when compared with control adults (Hall et al., 2010), which suggests a mechanism of DNA-chromatin reprogramming able to erase epigenetic marks.

Interestingly, we observed that the effect of testosterone in the gentle touch response was maintained, in the absence of the hormone, in the four subsequent generations after the exposition to the steroid. This observation suggests that the epigenetic effects of testosterone can be inherited transgenerationally. However, the results in the gentle touch contrast with the observed in the pharyngeal pumping rate, where the reduction rate induced by testosterone disappears in the next generation.

The molecular mechanisms underlying transgeneracional epigenetic marks are insufficiently understood (Youngson and Whitelaw, 2008). Research in *C. elegans* has illustrated several cases of transgenerational epigenetic inheritance. For example, it has been reported that modified histones incorporated into the sex chromosomes during spermatogenesis persist for several cell divisions postfertilization (Bean et al., 2004). One possible explanation for the differences between the gentle touch response and pharyngeal pumping rate after the exposure to testosterone is that the removal of epigenetic marks between generations depends on their position in the genome. Regarding this idea, a recent study demonstrates that deficiencies in genes involved in the COMPASS complex can induce transgenerational inheritance of longevity (Greer et al., 2011). The COMPASS complex, conserved from yeast to humans, catalyzes the trimethylation of histone H3 at lysine 9 (H3K4me3). H3K4me3 histone marks are usually present at the promoters of actively transcribed genes (Barth and Imhof, 2010). But the most remarkable phenomenon was that genetically wild type descendants from ancestors with a mutation in the COMPASS complex still display extended lifespan until the third generation (Greer et al., 2011). Interestingly, the number of genes showing down differential regulation was 7820 and 1740 genes in P0 and F4 generations respectively, in respect of the wild type strain. This observation suggests that the transgenerationally inherited gene misexpression profile returned to normal at the next (F5) generation, suggesting that these phenotypes may be directly linked. Thus, in this case the H3K4me3 dearth would be replenished, but only progressively, at each generation, until the chromatin state near the key mediator genes responsible for the longevity phenotype could be completely reset (Benayoun and Brunet, 2012). A similar mechanism could explain the differences between the gentle touch response and pharyngeal pumping rate phenotypes in successive generations after the exposure to testosterone.

Other potential molecular mechanism underlying the transgenerational inheritance could be activated by the production and transmission of non-coding RNAs. The introduction of double-stranded RNA (dsRNA) triggered sequence-specific genetic interference (RNAi) that was transmitted to offspring with the single sperm and maintained for three or more generations (Grishok et al., 2000; Grishok, 2013 and references therein). To this respect, genomic analysis demonstrated a connection between RNAi, chromatin and transgenerational RNAi inheritance (Gu et al., 2012). This work identified dsRNA-induced histone H3 lysine 9 trimethylation (H3K9me3) marks at several loci in animals fed with dsRNA. These H3K9me3 marks were also present in the F1 and F2 progenies, and they could spread up to 11 kb away from the dsRNA trigger region (Gu et al., 2012). On other hand, it was shown that a germline nuclear small RNA/chromatin pathway can maintain stable inheritance for many generations when triggered by a piwi-interacting RNA (piRNA)-dependent foreign RNA response (Ashe et al., 2012). There is a recent report that relates testosterone, epigenetic, and miRNA function (Morgan and Bale, 2011).

In conclusion, our results demonstrate that testosterone can influence the behavior of *C. elegans*. The hormone appears to act causing epigenetic marks that can be inherited transgenerationally. Due to the finding of a positive association between elevated levels of fetal testosterone and autistic traits in humans, these results found in the nematode may be relevant to the understanding of mechanisms by which the hormone may interact with the nervous system.

### Conflict of interest statement

The authors declare that the research was conducted in the absence of any commercial or financial relationships that could be construed as a potential conflict of interest.
